# *In* *-* *vitro* Assessment of the Antiproliferative and Apoptotic Potential of the Ethyl acetate Extract of *Peltophorum* *africanum* on Different Cancer Cell Lines

**Published:** 2017

**Authors:** Benjamin Ifeoluwa Okeleye, Noxolo Thabiso Mkwetshana, Roland Ndip Ndip

**Affiliations:** a *Microbial Pathogenicity and Molecular Epidemiology Research Group, Department of Biochemistry and Microbiology, Faculty of Science and Agriculture, University of Fort Hare, South Africa. *; b *Phytomedicine and Phytopharmacology Research Group, Department of Plant Sciences, University of the Free State, Qwaqwa Campus, Phuthaditjhaba 9866, South Africa. *; c *Department of Microbiology and Parasitology, Faculty of Science, University of Buea, Box 63, Buea, Cameroon.*

**Keywords:** *Peltophorum africanum*, Medicinal plant, Antiproliferation, apoptosis, Chemotherapy

## Abstract

We evaluated the *in-vitro* antiproliferative and apoptotic potential of the ethyl acetate extract (EAE) of *Peltophorum africanum*, a member of the family Fabaceae (Sond) in order to validate its pharmacological use. Antiproliferation of human breast (MCF-7), colon (HT-29) and cervical (HeLa) cancer cell lines by EAE was investigated using the Cell Titer-Blue viability assay and the mechanism of action delineated using the Nucleic Acid and Protein Purification Nucleospin^®^ Tissue Kit, Scanning Electron Microscope (SEM), propidium iodide (PI) and acridine orange (AO) double-staining techniques. We observed a significant reduction in cell viability of the MCF-7 cells from 100% (untreated) to 54.33 ± 1.84% after 72 h of treatment with 5 µg/mL of EAE (*P. value* < 0.05). Internucleosomal DNA of MCF-7, HT-29 and HeLa cells was randomly fragmented into an uninterrupted spectrum of sizes, complemented by the intercalation of nucleic acid-specific fluorochromes by PI and AO spotting two phases of apoptosis; early (EA) and late (LA) apoptosis. Distinctive ultramorphological changes observed included; cell shrinkage, membrane blebbing, and typical cell induced death. The ethyl acetate extract of *P.africanum* has the potential to induce apoptosis and is undergoing further studies *in-vivo* as a likely template for new anticancer therapy.

## Introduction

Cancer is the uncontrolled growth of cells coupled with malignant behavior: invasion and metastasis; resulting from genetic and environmental interactions (1). It has been reported that about 80-90% of all cancers is associated with environmental factors, and about 35% of them may be due to the effects of dietary factors (2). More than 10% of all deaths worldwide are caused by cancers. The breast cancer among women in India range from 25 to 31 cases per 100,000 (3). Cervical cancer is the most common cancer in Thailand with the age standardized incidence rate (ASR) of 19.5 per 100,000 person-years (4). Preserved and red meat is directly linked to an increased risk for colorectal cancer (5). 

Acute myeloid leukaemia and other metastases have been reported to result from the drugs such as irinotecan used in the treatment of other cancer (6, 7). Over time, cancer cells become more resistant to chemotherapy treatments; recently small pumps on the surface of cancer cells that actively pump drugs from inside the cell to the outside were identified (1, 8). Momentous research efforts have focused on novel chemotherapeutic drugs from medicinal plants in search of cancer inhibitors and therapy (9). Such plants are valuable sources of bioactive compounds and phytochemicals with various bioactivities, including antioxidant, anti-inflammatory and anticancer activities which play a major role in human health care, as about 65 % of the world population relies on the use of alternative medicines (10). 

*P. africanum*, a locally used medicinal plant in the treatment of colic, diarrhoea, human immunodeficiency virus/acquired immune deficiency syndrome (HIV/AIDS), and infertility common in South Africa has been widely studied for its cytotoxicity, antioxidant and antimicrobial, with profound activities reported (11). To the best of our knowledge, the anticancer activity of this plant has not been evaluated. In view of this, we therefore aimed at investigating the antiproliferative effect of the ethyl acetate extract of the plant on human breast, cervical and colon cancer cell lines and its likely mechanisms of actions, in an effort to find a relatively cheap source of bioactive lead which could serve as a useful template for the synthesis of an anticancer agent. 

## Experimental

*Preparation of the extract*

*Peltophorum africanum* (stem bark) was selected based on our previous study. The plant was collected, identified and archived in Limpopo Province, South Africa in collaboration with a botanist at the University of Venda (voucher specimen number BP01). The extraction and the sterility test were done as previously reported (11). Briefly, dried powder of the plant part was extracted with ethyl acetate and filtered after 48 h. The plant residue was re-extracted exhaustively and the filtrate was concentrated on a rotary evaporator (Strike 202 Steroglass, Italy) at 70 ^O^C to take out the ethyl acetate. Working stock of the extract was prepared by sterilizing in 100% DMSO for each bioassay analysis. The extract was aseptically bottled using Acrodisc 25 mm PF Syringe (Pall, USA), tested for sterility and then stored at 4 ˚C before use. Ethyl acetate extract (EAE) was selected for this study because of its cytotoxic potency against normal human liver cell and marked antimicrobial activity as reported in our previous study (11). 

*Cancer cell culture and maintenance*

Cancer cell lines; MCF-7 (breast), HT-29 (colon) and HeLa (cervical) used in this study was a kind donation from Prof. Maryna Van De Venter of Nelson Mandela Metropolitan University, South Africa. Briefly, a vial containing cells (1 mL) was thawed in a water-bath regulated at 37 ˚C for 2 - 5 minutes and diluted with 9 mL pre-warmed (10 – 15 minutes in water bath at 37 ˚C) Dulbecco’s minimum essential medium (DMEM) containing 10 % fetal bovine serum (FBS). The cells were incubated in a 37 °C humidified incubator (Shel Lab, USA) at 5% CO_2_ for multiplication and adherence. Maintenance of cells was achieved as the old medium was aspirated, washed with 10 mL phosphate buffer saline (PBS), trypsinized (0.5 - 1 mL trypsin) and then split into a fresh medium until the desired cell number and confluence was attained. 

*Cell Titer-Blue viability assay*

Anticancer activity of the EAE of *P. africanum* was evaluated on three cancer cell lines (MCF-7, HT-29 and HeLa cell) using the microculture cell titer blue viability assay as previously described (Promega, USA) (12, 13). The 96-well microplates were seeded with 200 μL DMEM + high glucose, L-glutamine and sodium pyruvate (Thermo Sceintific, South Logan, Utah) containing 6.0 x 10^3^ cells in suspension and incubated in CO_2_ (5%) incubator at 37 ^o^C. After 24 h incubation and attachment, the cells were treated with 1000, 500, 250, 125, 75, 25 and 5 μg/mL concentration of the extract. Each plate included untreated cell controls and a blank cell-free control. Exactly 60 μM (previously reported IC_50_ value) of curcumin (Sigma-Aldrich, South Africa) was used as positive control and 0.5% (residual after dilution) DMSO as negative control. After 24, 48 and 72 h of incubation, cell viability was determined by adding cell titer blue as an indicator and further incubated for 4 h. Fluorescence was read at 570/620 nm using Analytical & Diagnostic Product Gen™ spectrophotometer (BioTek, Highland Park, USA). All experiments for the extract were carried out in triplicates and the concentration which inhibited 50% of cellular growth (IC_50_ value) was determined. 

*DNA fragmentation analysis*

Fragmentation of the DNA was analyzed in line with a previously described method (14). The cell lines; MCF-7, HT-29 and HeLa cell at a concentration of 1 × 10^6 ^mL^-1 ^each, were treated with EAE in a 6-well tissue culture plate after 24 h of attachment at IC_50,_ IC_50_ × 2, IC_50_ × 4 and IC_50_ × 8 (in duplicate), including a negative control (untreated cells) and positive control using previously reported IC_50_ value (60 µM curcumin and 10 µg/mL actinomycin D which is widely used in clinical practice since 1954 as an anticancer drug for treating many tumours with two main mechanisms of its action reported: intercalation to DNA and the stabilization of cleavable complexes of topoisomerases I and II with DNA). After 24 h of treatment, cells were harvested using sterile scraper and washed with PBS prior to DNA isolation. DNA extraction was carried out using Nucleic Acid and Protein Purification Nucleospin® Tissue Kit (Macherey-Nagel, Germany) according to the manufacturer’s manual instructions. Briefly, 10 µL of the DNA in TEA buffer was loaded onto 1.8% agarose gel containing 0.5 µg/mL (5 µL in 100 mL of gel) ethidium bromide. Electrophoresis was conducted progressively at 35, 67 and 100 V for 4 h and the DNA fragments were visualized and photographed under UV illumination XD – 79, WL/26 MX, 230 V ~ 50/60 HZ (Alliance 4.7, Taiwan, France). Based on the results obtained, we decided to proceed with analysis of only the MCF-7 cell line. 

*Determination of apoptosis using intercalation of nucleic acid-specific fluorochromes*

Morphological changes resulting in the leakage and fragmentation of DNA by EAE were assessed under fluorescence microscope using propidium iodide (PI) and acridine orange (AO) double-staining according to the method of Mohan *et al*. (15) with few modifications. MCF-7 cells were seeded in a 6-well tissue culture plate at a concentration of 1 × 10^6 ^mL^-1 ^and treated after 24 h with EAE at IC_50, _IC_50_ × 4 and IC_50_ × 8 respectively. Untreated cells were used as a negative control and cells treated with 60 µM of curcumin as a positive control. The experiment was performed in duplicate. The cells were collected after 24 hrs of treatment using a sterile scraper, centrifuged at 300 × g for 10 min and the cellular pellet washed twice with PBS. Fluorescent dyes; PI (10 µL) and AO (10 µL) were added to the cell (20 µL) at equal volumes and promptly observed under UV-fluorescence microscope. The percentages of viable, early apoptotic, late apoptosis and secondary necrotic cells were determined (15). 

*Morphological characterization of apoptosis*

The morphological characteristics of the cells (MCF-7) were determined using scanning electron microscope (SEM; JSM-6390LV, Jeol, Japan). Approximately 1 × 10^6 ^mL^-1 ^of the MCF-7 cells were seeded in a 6-well tissue culture plate and treated after 24 h with EAE at IC_50, _IC_50_ × 4 and IC_50_ × 8. Untreated cells and cells treated with 60 µM of curcumin were included as negative and positive control respectively. Cells were collected, washed twice in phosphate buffered saline (PBS) and then centrifuged at 1000 rpm for 5 min; after which it was fixed in 2.5% gluteraldehyde prepared in 0.1 M PBS. After washing, the cells were post fixed on poly-L-lysine-coated glass coverslip, with 1 % osmium tetroxide (OsO_4_) in 0.2 M PBS for 30 min, and then washed with PBS. Cells were mounted onto stubs and coated using IB3 Ion Coater (EIKO, Japan) after dehydrated through graded ethanol (30, 50, 70, 85 and 95%) and critical point dry (CPD). Different sections of the cells were micro-analyzed and the representative spectra presented (15, 16). 

*Statistical analysis*

Statistically significant differences among the three cell line and the apoptosis phase percentage values of MCF-7 compared with the control were determined by one way analysis of variance (ANOVA) and *P. value* < 0.05 was considered significant, while IC_50_ was determined using the regression analysis test. All analyses were performed with Minitab statistical software (student version 12 for windows). 

*Ethical consideration*

This study which is a continuation of our line of studies on antimicrobial activity and cytotoxicity of medicinal plants had been approved by the institutional review board of the University of Fort Hare, Alice, South Africa. 

## Results

*Anti-proliferation activity of the extract (EAE) against the cancer cell lines*

**Figure 1:**
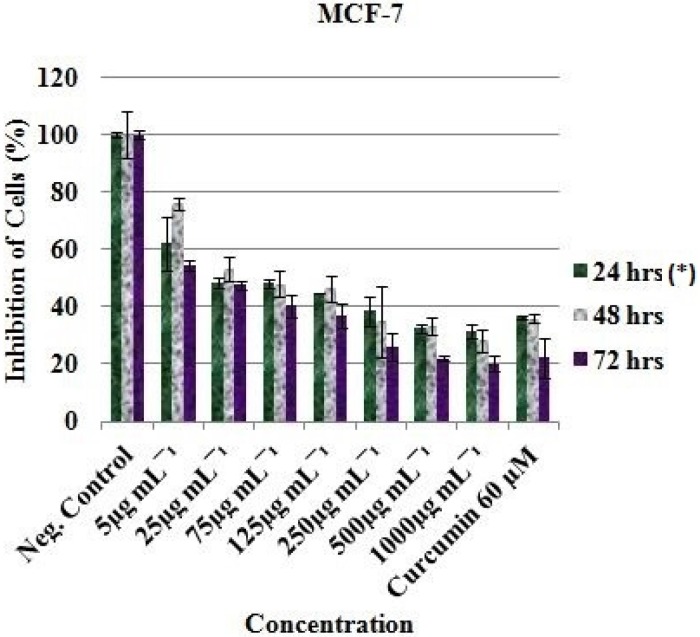
*In-vit* *r* *o* inhibitory profile of the EAE against MCF-7 cells. *Hours of treatment

The ethyl acetate extract (EAE) exhibited inhibitory effect against the MCF-7, HT-29 and HeLa cell lines. The viable cell of MCF-7 was significantly reduced from 100% (Negative control) to 62.20 ± 9.41 %, 75.92 ± 2.14 % and 54.33 ± 1.84 % after treatment with 5 µg/mL of EAE at 24, 48 and 72 h respectively (*P. value* < 0.05). Meanwhile reduction of viable cell to 32.55 ± 1.55 %, 33.11 ± 2.92 % and 22.00 ± 0.80 % were noted after treating with 500 µg/mL of EAE at 24, 48 and 72 h respectively (Figure 1). 

**Figure 2:**
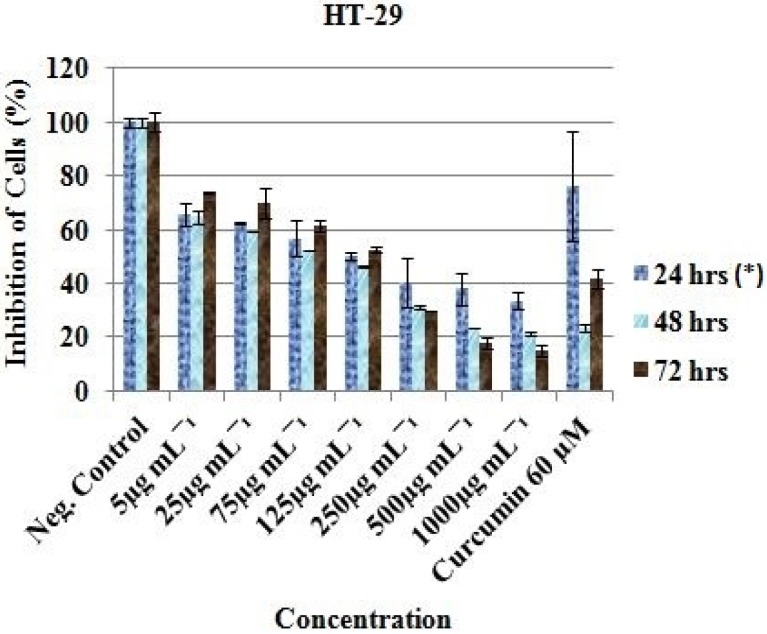
*In-vit* *r* *o* inhibitory profile of the EAE against HT-29 cells. *Hours of treatment

At 5 µg/mL - 1000 µg/mL, the HT-29 cell proliferation decreased from 65.74 ± 4.36%, 64.75 ± 2.23% and 73.82 ± 0.27% to 33.57 ± 3.17%, 21.21 ± 0.57% and 14.80 ± 2.21% after 24, 48 and 72 h respectively (Figure 2). 

**Figure 3:**
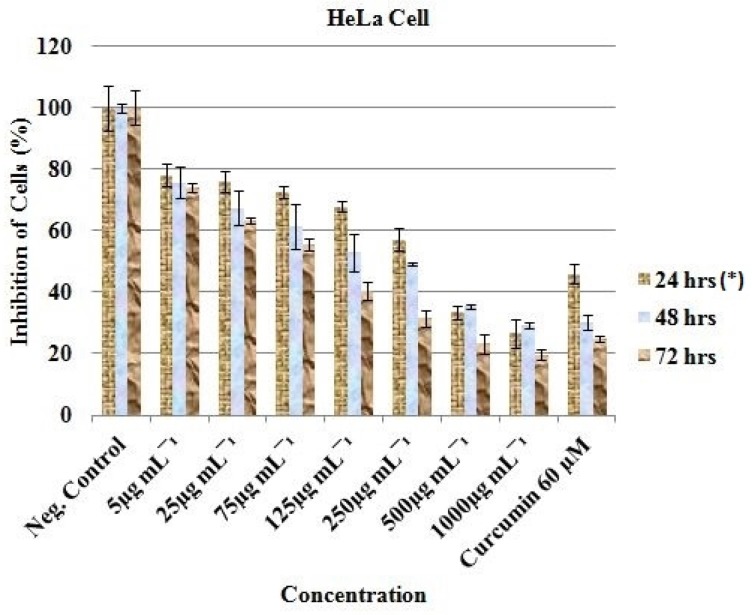
*In-vit* *r* *o* inhibitory profile of the EAE against HeLa cells. *Hours of treatment

For HeLa, decline in viable cells ranged from 78.22 ± 3.71 (5 µg/mL, 24 h) to 19.74 ± 1.60 (1000 µg/mL, 72 h). The percentage reduction in proliferation of all the cancer treated cell lines was conspicuous and statistically significant (*P. value* < 0.05; Figure 3). 

Curcumin (Positive control) was observed to be active at 60 µM as the cells were reduced from 100% (Negative control) to 36.15 ± 0.60 (24 h), 35.93 ± 1.57 (48 h) and 22.23 ± 6.86 (72 h) for MCF-7 (Figure 1); 76.44 ± 20.34 (24 h), 23.48 ± 1.70 (48 h) and 41.78 ± 3.34 (72 h) for HT-29 (Figure 2); and 45.99 ± 3.32 (24 h), 30.19 ± 2.58 (48 h) and 24.98 ± 0.93 (72 h) for HeLa cell (Figure 3). Inhibitory concentration at 50 % (IC_50_ in µg mL^-1^) of MCF-7 (IC_50_ = 389.05 ± 7.56, 24 h; 312.84 ± 6.55, 48 h; 321.38 ± 5.91, 72 h), HT-29 (IC_50_ = 281.16 ± 4.389, 24 h; 331.19 ± 5.67, 48 h; 330.86 ± 8.56, 72 h) and HeLa cell line (IC_50_ = 310.16 ± 6.33, 24 h; 375.0 ± 4.71, 48 h; 325.23 ± 8.03, 72 h) were recorded using regression analysis. 

*Internucleosomal DNA fragmentation*

**Figure 4:**
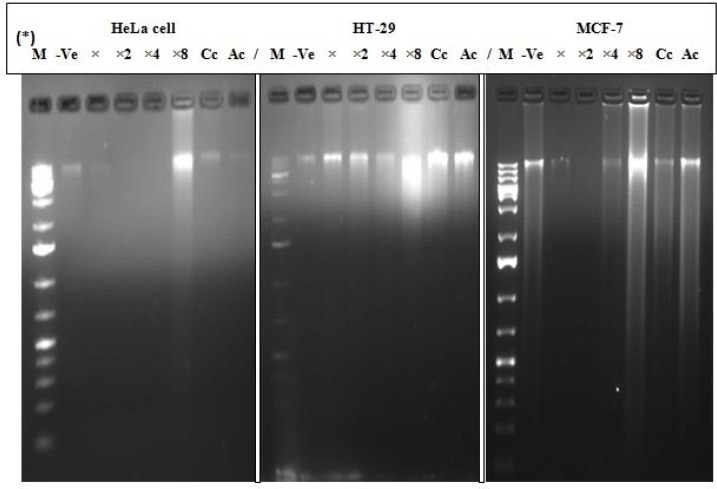
Cellular degradation and random fragmentation of DNA. *M = Marker, -Ve = Negative control (untreated cell), X = IC_50_ , X2 = IC_50_ ×2, X4 = IC_50_ ×4, X8 = IC_50_ ×8, Cc=Curcumin (60 µM), Ac = Actinomycin D (10 µg/mL

The DNA of the treated MCF-7, HT-29 and HeLa cell lines exhibited a random degradation with a non-specific and continuous spectrum of sizes during the cellular degeneration after 24 h treatments. Prominent random fragmentation of the DNA was observed at IC_50_×8 for all the three treated cells (MCF-7, HT-29 and HeLa) compared to the positive control (curcumin and actinomycin D) indicating early apoptosis. Moderate degradation was observed at IC_50_, IC_50_×2 and IC_50_×4 for HT-29 and IC_50_×4 for MCF-7 signifying late apoptosis (Figure 4). 

*Phases and degree of apoptosis*

**Figure 5:**
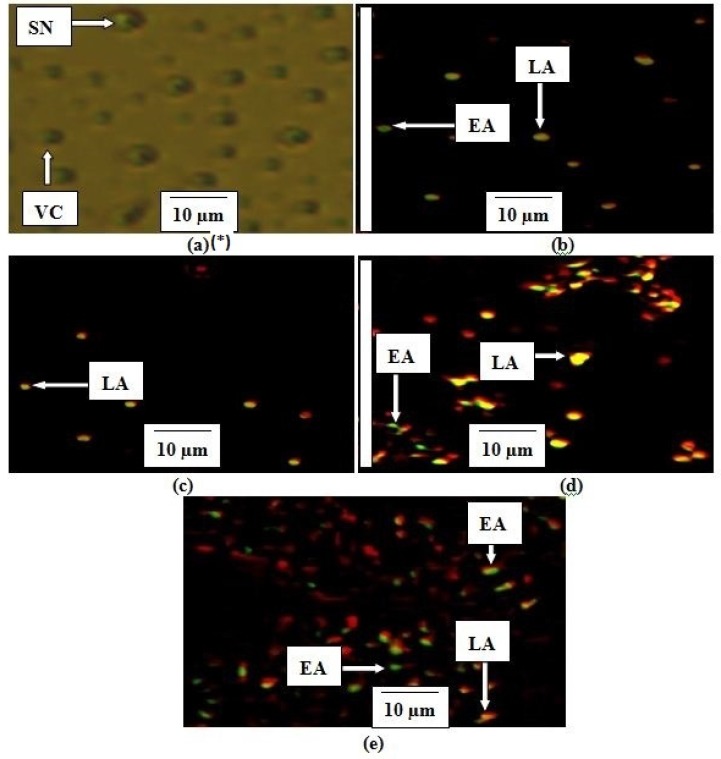
Stages of apoptosis in MCF-7. *(a) Untreated cell (negative control), (b) Curcumin (60 µM), (c) EAE IC_50_ , (d) EAE IC_50_ ×4, (e) EAE IC_50_ ×8. The criteria for identification: 1. Viable cells (VC) observed to have green normally shaped nuclei, 2. Early apoptosis (EA) appeared to have light-green nucleus showing aggregation and condensation of chromatin, 3. Dense orange area of chromatin condensation showing late apoptosis (LA) and 4. Orange intact nucleus portraying secondary necrosis (SN

**Figure 6:**
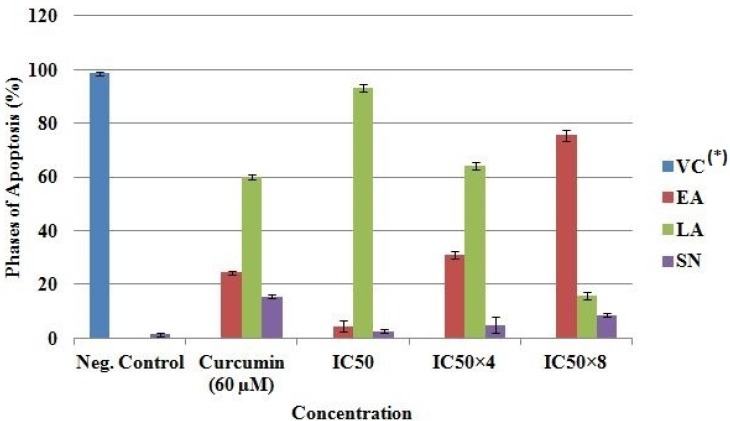
Quantification of stages of apoptosis in MCF-7. *VC, Viable cell; EA, Early apoptosis; LA, Late apoptosis; SN, Secondary necrosis

Propidium iodide (PI) and acridine orange (AO) double-staining analysis revealed phases of morphological changes and the degree of induction of apoptosis. Intercalation of AO within the fragmented DNA was observed in early apoptosis (EA) as bright-green colour in treated cells (MCF-7) compared to untreated cells with a green normal structure (Figure 5). Approximately 75.5 ± 2.1, 31 ± 1.4, 24.4 ± 0.7 and 4.5 ± 2.1% expression of EA were observed in IC_50_×8, IC_50_×4, curcumin and IC_50 _respectively (Figure 6). Late apoptosis (LA) intercalated PI and AO, appeared as reddish orange and secondary necrosis (SN) as orange nucleus with intact structure (Figure 5). 

The expression of LA was 93 ± 1.4, 64 ± 1.4, 60 ± 0.9 and 16 ± 1.4% for IC_50_, IC_50_×4, curcumin and IC_50_×8 respectively. Apoptosis (EA) of MCF-7 increased significantly (p < 0.05) in a concentration dependent manner; however, no significant (p > 0.05) difference was observed in the SN cell (Figure 6). 

*Ultrastructural characterization of treated cells*

Distinctive morphological changes corresponding to characteristic cellular apoptotic stages were observed in SEM analysis with the degree and the rate of morphological changes become greater as extract concentrations increased, reaching the maximal (typical apoptosis) responses at IC_50_×8 of EAE. Visible changes observed include, decrease in cell volume, cell shrinkage, cell retraction, formation and separated of apoptotic bodies, membrane blebbing and typical cell induced death 

(Figure 7). 

**Figure 7:**
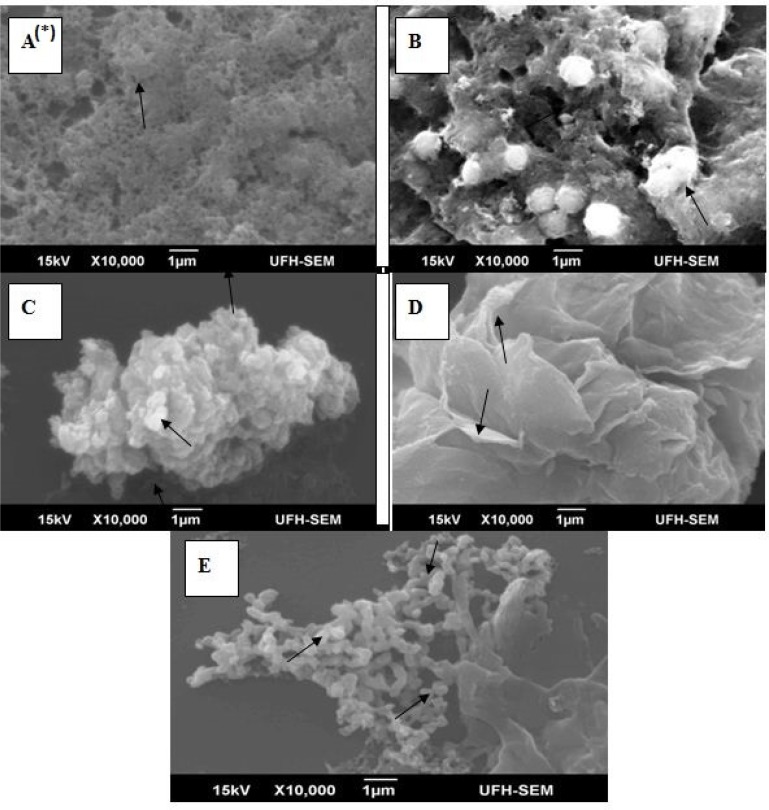
Ultrastructural changes of apoptosis in MCF-7. *(A) The characteristic of untreated cells was observed as flat and with smooth surface, a typical morphological feature of a cancer cell; (B) Curcumin treated MCF-7 (60µM), showed different form of rounding and cell shrinkage; (C) EAE treated MCF-7 (IC_50_ ) demonstrated cell shrinkage, membrane blebbing and separated apoptotic bodies; (D) EAE treated MCF-7 (IC_50_ ×4) exhibited surface blebbing and cell retraction and; (E) EAE treated MCF-7 (IC_50_ ×8) showed signs of typical apoptosis (induced cell death

At IC_50_, EAE treated MCF-7 extensively demonstrated cell shrinkage, membrane blebbing and separated apoptotic bodies (Figure 7 c) compared to flat, smooth and confluent negative control (Figure 7 a). 

## Discussion

Apoptosis is a highly regulated systematic form of programed cell death, functioning as a regulator of biological homeostasis; often associated with the cells that are advancing towards the cell cycle (17). It is an energy consumed process involving loss of cell to cell contact, cell shrinkage, condensation of nuclear chromatin, and finally endonucleolytic fragmentation of genomic DNA (18). Necrosis on the other hand is initiated by severe toxic stress, characterized by deterioration of ATP concentrations, intracellular damage of organelles and induction of an acute inflammatory response (19). Apoptosis has been reported to be initiated by natural products which are similar to the observations noted in the current study (11, 12, 14, 20, 21, 22). 

Cell proliferation analysis using CellTiter-Blue cell viability assay revealed notable apoptotic effect of ethyl acetate extract (EAE) of *P. africanum* in a concentration dependent manner against human breast (MCF-7), colon (HT-29) and cervical (HeLa) cancer cell. Exactly 25 µg/mL of EAE reduced MCF-7 viable cell to 48.38 ± 1.56% after 24 h of treatment. Approximately 62.36 ± 0.42 and 76.10 ± 3.54 % reductions were observed against HT-29 and HeLa cells respectively, at the same concentration and time of treatment. This is similar to the study conducted by Lee *et al*. (20) who reported water extract of *Strychni Semen* (ESS) to inhibit the growth of AGS human gastric carcinoma cells. 

We have demonstrated that the treatment of MCF-7, HT-29 and HeLa cancer cells with EAE caused alteration in genetic material (DNA fragmentation) and morphological changes showing a random degradation with a continuous spectrum of sizes accompanying cellular degeneration. This corroborate the study of Fernandez *et al*. (19), he reported the induction of cell death of chicken embryonic fibroblasts *in-vitro* by addition of actinomycin D, an inhibitor of RNA polymerase and a cancer chemotherapeutic agent. The nuclear and cytoplasmic condensation of the cells *in-vitro* was observable 2 to 3 h after treatment and genomic DNA isolated from such cells showed typical DNA fragmentation patterns. Internucleosomal fragmentation of genomic DNA which has been reported as one of the leading biochemical markers of apoptosis (23, 24) results in the production of oligonucleosomal fragments of different molecular weight which is generated by chromatin and enhanced by the Ca/Mg-dependent endonuclease cascade. Though we did not investigate the role of caspase 3 in the current study (currently receiving attention in our group), it has been reported that DNA fragmentation is set off by caspase 3 activation of inactive CAD (caspase activated deoxyribonuclease) through exclusion of its inhibitors; a biochemical characteristic of inherent apoptotic cell death (25, 26). This might have been responsible for our observed changes. 

Breast cancer is one of the most common cancers in women with an increasing global prevalence (27). Cervical cancer is considered a critical public health problem; second to breast cancer in women worldwide (28, 29). As shown by the SEM analysis, EAE-treated MCF-7 breast cancer cell lost its flattened morphology, became shrunk with membrane blebbing and separated apoptotic bodies due to cytoplasm retraction around the nucleus compared to the negative control that still appeared flat and well attached to the substrate. This is similar to the observations reported by other investigators (13, 30). Cytological observations by AO and PI double staining and SEM analysis further showed multinucleation, holes and abnormalities of mitochondrial cristae of the apoptotic cells. 

Cell-based lethality is an indication of bactericidal, fungicidal and cytotoxicity activities. The IC_50_ value obtained in this study is in line with the cytotoxicity values reported by Okeleye *et al*. (11) which indicates that the EAE has high pharmacological actions. The activity observed in EAE is ascribed to the compounds identified which are reported in our previous study. For example, palmitic acid or n-hexadecanoic acid, is a saturated fatty acid. Many fatty acids are known to have antinflamatory, antibacterial and antifungal properties, and can modulate immune responses by acting directly on T cells (11). Stigmasterol is used as a precursor in the manufacture of vitamin D3 and semisynthetic progesterone, a valuable human hormone that plays an important physiological role in the regulatory and tissue rebuilding mechanisms. Campesterol molecules compete with cholesterol and thus reduce the absorption of cholesterol in the human intestine. Campesterol has anti-inflammatory effects and is mostly used as a treatment for benign prostate hyperplasia (9-11, 13). Phytosterols are recognized as save ingredients that lower blood cholesterol. Gallic acid, Lupeol, ferulic acid, and Hop-22(29)-en-3.beta.-ol helps to protect human cells against oxidative damage and acts as an antioxidant, anticancer anti-inflammatory and antiulcerogenic agent (11). 

A continuous spectrum of DNA fragmentation was observed in this study as opposed to a distinct internucleosomal DNA cleavage. Extracellular Ca^2+^ has been reported to enhance internucleosomal DNA cleavage (23, 24, 31). Reports in the literature holds that compounds which protect cells or elevate K^+^ suppresses DNA fragmentation while any agent(s) which activates a variety of cellular protein kinases (PKC) are capable of supporting cell survival, but not necessarily needed to inhibit internucleosomal DNA fragmentation (32). However, zinc significantly inhibits specific internucleosomal and nonspecific DNA fragmentation (33, 34). A loss of intracellular Cl^-^ is normally associated to cell volume reduction, which is a late apoptotic event common to most apoptotic pathways. A high extracellular NaCl medium creates an unfavourable electrochemical gradient for Cl^-^ efflux and could alter the cytosolic ionic composition that inhibits nucleosomal DNA cleavage during the apoptotic process (35); Cl^-^ efflux coupled with the activation of K^+^ channels sustains electroneutrality, particularly when cytosolic volume diminishes. Obstruction of a class of K^+^ pathway has been reported to deter apoptosis associated cell shrinkage; hence, demonstrating the related mechanisms of cell contraction in late apoptotic phases (36). In a separate study (data unpublished), we demonstrated the presence of several metals/ions in this extract, which had a remarkable effect on the morphology of bacterial and yeast cells. Bearing in mind that most known proteins contain metal (Na^+^, K^+^, Mg^2+^, Ca^2+^, Zn^2+^, Cu^2+^, Fe^2+^, Co^2+^, and Mn^2+^) cofactor(s), which carry out an array of tasks ranging from protein structure stabilization to enzyme catalysis, activating many fundamental life processes (37) we are constrained to speculate that these ions could have been responsible for the observed continuous spectrum of DNA fragmentation as opposed to specific or distinct DNA fragmentation in line with the cited reports. 

Previous studies have reported that defects in apoptotic pathways or repression of apoptosis contribute to expansion of cancer development (38). Natural products that are able to eliminate abnormal cells by induction of apoptosis rather than inhibit or repress malignant growth; that are less toxic and mutagenic than current treatment regimens, will produce new therapies against cancer chemoprevention and cure (39, 40). With the multitude of compounds reported to be present in the EAE of *P*.* africanum* in our previous study (11); it is therefore probable that a mixture of therapeutic agents are needed for utmost therapeutic benefits. 

## Conclusion

This study demonstrated that ethyl acetate extract (EAE) significantly inhibited the proliferation of MCF-7, HeLa and HT-29 treated compared to untreated cells. However, a stronger cytotoxic effect on MCF-7 cell was noted through ultrastructural disruption and early internucleosomal DNA fragmentation via membrane leakage; indicating its valuable marker for investigating the mechanisms of cell viability and apoptosis. The current findings therefore call for further studies using animal models to investigate possible antitumor leads from ethyl acetate extract of *Peltophorum africanum*. 
